# Mobile resistome of microbial communities and antimicrobial residues from drinking water supply systems in Rio de Janeiro, Brazil

**DOI:** 10.1038/s41598-022-21040-7

**Published:** 2022-11-09

**Authors:** Kayo Bianco, Beatriz Oliveira de Farias, Andressa Silva Gonçalves-Brito, Ana Paula Alves do Nascimento, Mariana Magaldi, Kaylanne Montenegro, Claudia Flores, Samara Oliveira, Mychelle Alves Monteiro, Bernardete Ferraz Spisso, Mararlene Ulberg Pereira, Rosana Gomes Ferreira, Rodolpho Mattos Albano, Alexander Machado Cardoso, Maysa Mandetta Clementino

**Affiliations:** 1 Instituto Nacional de Controle de Qualidade Em Saúde INCQS/FIOCRUZ, Oswaldo Cruz Foundation, Rio de Janeiro, RJ 4365 Brazil; 2 Rio de Janeiro State University, Rio de Janeiro, Brazil

**Keywords:** Antimicrobials, Microbial communities, Environmental microbiology

## Abstract

Antibiotic resistance genes (ARGs) are widespread in the environment due to the overuse of antibiotics and other pollutants, posing a threat to human and animal health. In this study, we evaluated antimicrobial residues, bacterial diversity and ARGs in two important watersheds, Guandu and São João, that supply drinking water to Rio de Janeiro city, Brazil. In addition, tap water samples were collected from three different cities in Rio de Janeiro State, including the metropolitan area of Rio de Janeiro city. Clarithromycin, sulfamethoxazole and azithromycin were found in untreated water and drinking water in all samples. A greater abundance of Proteobacteria was observed in Guandu and São João watersheds, with most of the sequences belonging to the Gammaproteobacteria class. A plasmidome-focused metagenomics approach revealed 4881 (Guandu), 3705 (São João) and 3385 (drinking water) ARGs mainly associated with efflux systems. The genes encoding metallo-β-lactamase enzymes (bla_AIM_, bla_GIM_, bla_IMP_, and bla_VIM_) were detected in the two watersheds and in drinking water samples. Moreover, we demonstrated the presence of the colistin resistance genes *mcr*-3 and *mcr*-4 (both watersheds) and *mcr*-9 (drinking water and Guandu) for the first time in Brazil. Our data emphasize the importance of introducing measures to reduce the disposal of antibiotics and other pollutants capable of promoting the occurrence and spread of the microbial resistome on aquatic environments and predicting possible negative impacts on human health.

## Introduction

The environmental impacts that most affect the quality of aquatic ecosystems and, consequently, public health are strongly associated with inadequately treated or untreated wastewater^1,2^. Water pollution can occur due to a lack of sanitation and/or waste discharge without treatment by point or diffuse sources^3,4^.

Several substances have been considered emerging contaminants, including new pesticides, antimicrobials, personal care products, some by-products from water disinfection processes, sweeteners such as sucralose, nanomaterials, and some microorganisms^5,6^. Recent estimates indicate that antimicrobials are the main classes of drugs capable of causing some of the greatest environmental impacts^7^.

Antimicrobials have been widely used in human and veterinary medicine. However, about 70 to 80% of ingested doses are excreted unchanged and discharged to water bodies, mainly through wastewater generated from hospitals and pharmaceutical industries^8^. These drugs are only partially removed by wastewater treatment and, depending on the compound, they can still be found at levels ranging between 10 and 1000 ng L^−1^ in effluents^9,10^.

The antimicrobials carried by effluent disposal in the environment, even at low levels, are a key signal that promotes gene dissemination and consequently increased resistance^11^. Many antimicrobials are naturally biodegradable compounds, but synthetic drugs such as quinolones are more resistant to biodegradation in the environment. This leads to prolonged effects on bacterial communities and to a substantial impact on increased resistance. Even when antimicrobial contamination is eliminated, the resistance determinants can be maintained and disseminated within and between microbial populations^12,13^.

Additionally, the disposal of antimicrobial residues in aquatic environments can not only cause impacts on the biodiversity and function of ecosystems but may also select for antibiotic resistant bacteria (ARB) and stimulate the dissemination of antimicrobial resistance genes (ARG)^14^. Mobile genetic elements (MGE), including phages, plasmids, and transposons, among others, mediate this spread^15^. Plasmids, in particular, are rapidly disseminated in the environment and play a major role in microbial evolution and adaptation as vehicles of gene transfer^16^.

The plasmidome is defined as the total plasmid populations within a given community^17^. Plasmidome analysis provides information on the composition and structure of the mobile resistome. Therefore, it is considered a promising approach that provides information about the types of plasmids present in the studied microbial community, and the MGE contained in these plasmids^18^.

The role of non-clinical environments in increasing the spread of ARBs has not been fully elucidated. In general, ARGs are not easily removed from polluted areas, even when the selective pressure exerted by pollutants is gone. This may also explain why ARGs are often found in antimicrobial-free environments^19,20^. Resistance to antimicrobials, initially confined to hospitals, has also been observed in the natural environment, which raises great concern regarding the impacts on human health. ARBs and ARGs can be dispersed into raw sources of drinking water, mainly through discharge of human and animal waste, wastewater treatment plant, hospital sewage, and agricultural practices such as manure application^21,22^.

The present study evaluated the presence, distribution, and abundance of antimicrobial residues and ARGs in two important watersheds for drinking water supply to South Central regions of Rio de Janeiro state, Brazil, including the metropolitan region of Rio de Janeiro city, Brazil, using high-performance liquid chromatography coupled to tandem mass spectrometry and a culture-independent approach, respectively. Our study may provide relevant information about the structure, complexity, and content of the plasmidome of these waters, which can pose serious threats to human health.

## Results

### Antimicrobial detection

**Figure 1:**
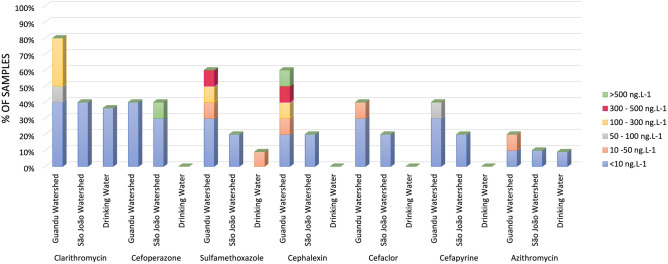
Detection frequency and concentration levels of antimicrobials in grouped samples of São João watershed, Guandu watershed, and Drinking water.

The samples were grouped as São João watershed, Guandu watershed, and Drinking water. Clarithromycin was the most frequently detected antimicrobial, being observed in 80% (8/10) of the Guandu samples, 40% (4/10) of the São João samples and 36% (4/11) of drinking water samples. In the first collection at São João River mouth, cefoperazone concentrations > 500 ng L^−1^ were found. The sulfamethoxazole concentration, belonging to the sulfonamide class, ranged from 47.4 to 340.5 ng L^−1^ in the second collection in the Macacos and Queimados rivers, respectively. The Unamar drinking water sample presented levels of 12.5 ng L^−1^ of this antimicrobial. Azithromycin was found in the drinking water samples from Itaguaí, in Macacos river and the São João river mouth at a concentration below 10 ng L^−1^, and 49.9 ng L^−1^ in the second collection in the Queimados river. Troleandomycin and roxithromycin were detected only in the Leblon drinking water sample at concentrations < 10 ng L^−1^ (Fig. 1).

### Microbial diversity

**Figure 2:**
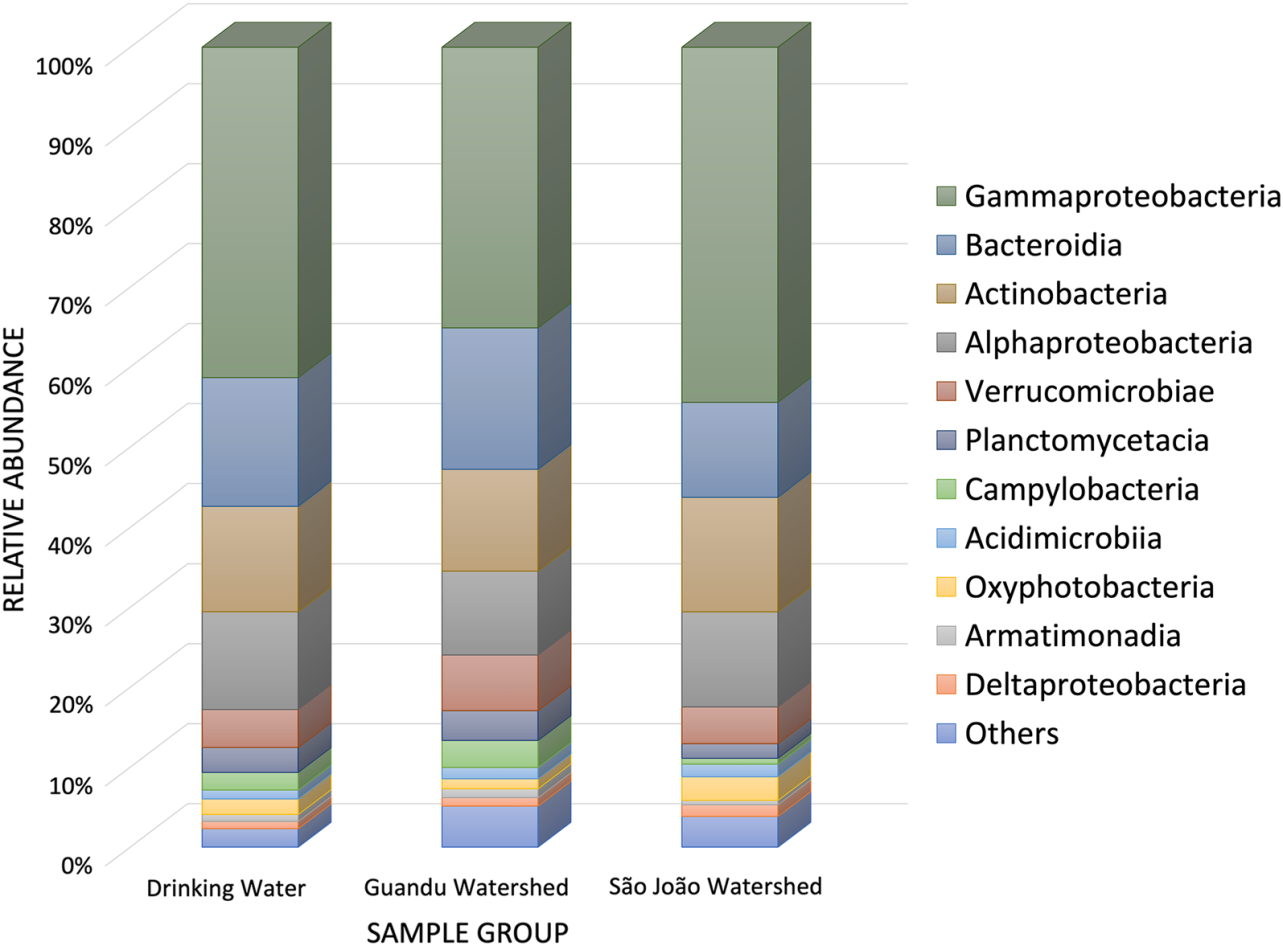
Relative abundance of bacterial composition at the class level in grouped samples of São João watershed, Guandu watershed, and Drinking water.

**Figure 3:**
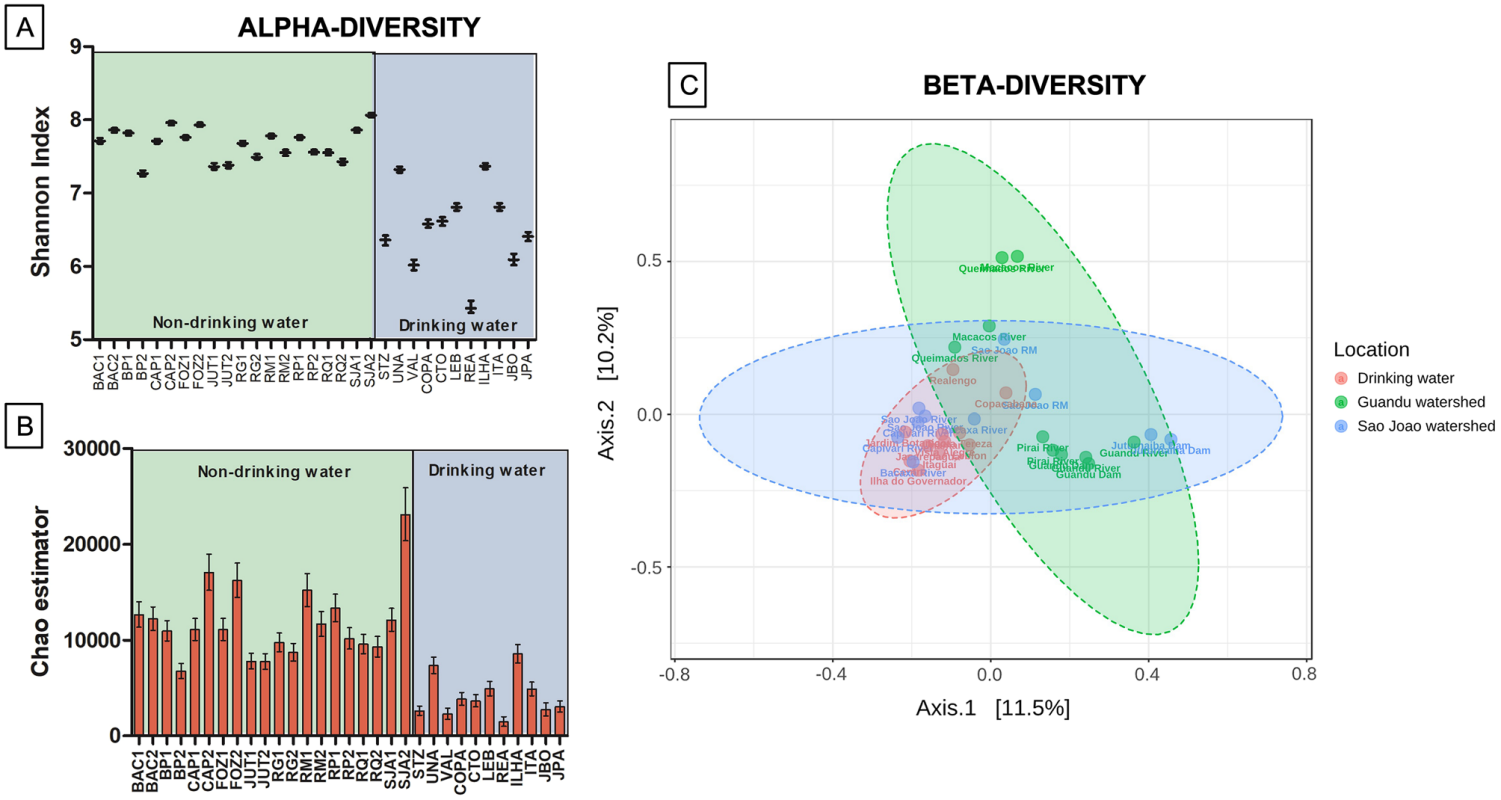
Diversity indices (Averages). (**A**) Comparison of the alpha diversity of the Operational taxonomic unit (OTUs) between the collection of untreated and treated water samples. Shannon coverage analysis; (**B**) Comparison of alpha diversity of bacterial OTUs among the analyzed water samples. Chao1 coverage analysis; (**C**) Principal coordinate analysis (PCoA) between bacterial communities present in the Guandu watershed (green), São João watershed (blue), and drinking water (red).

Nine bacterial phyla were observed, with *Proteobacteria* predominating in 93.5% (29/31) of the samples, followed by *Actinobacteria* and *Bacteroidetes*. Within the phylum *Proteobacteria*, the predominant class was *Gammaproteobacteria* (36 to 46%), followed by *Alphaproteobacteria* (11–13%). In the *Bacteroidetes* phylum, the *Bacteroidia* class was the most abundant (12–17%). In the *Actinobacteria* phylum, the class *Actinobacteria* was the most abundant (12–14%) (Fig. 2). There were no significant differences (*p* > 0.05) in the alpha diversity of microbial communities associated with the seasonality of sample collection. Therefore, the samples were grouped as São João watershed, Guandu watershed, and Drinking water (Fig. 3a).

The effect of sample types on alpha bacterial diversity was assessed based on the richness of OTUs (absolute number of taxa), diversity, and uniformity. The bacterial communities in the drinking water samples presented lower diversity, compared to untreated water samples from the watersheds (*p* < 0.02 with Shannon Index) (Fig. 3A and B). There were no statistically significant differences between these samples so they were pooled. The water samples also showed significant variations (*p* < 0.001) concerning the beta diversity of bacterial communities. Meanwhile, the samples did not show significant variations (*p* < 0.103) by the Jaccard method (Fig. 3C). We believe the wide variations observed between the samples reflect the environmental conditions that are very dynamic in these aquatic habitats, such as rainfall, and sewage discharge, for example. As our main goal was to determine antimicrobial concentrations and the presence of ARGs in the plasmidome in these samples, we believe there is no need to collect more samples. More detailed Alpha diversity measures are presented in supplementary material Table S1, revealing that bacterial community richness (Chao1), diversity (Shannon and Simpson), and evenness (Shannon even) varied widely among the samples.

### Plasmidome analyses

A total of 3,490,453 paired-end reads were generated for São João watershed, 2,719,506 for Guandu and 3,302,359 drinking water samples. After quality control and assembly, 6197 contigs from the São João watershed (mean sequence length 2.043 bp), 4866 from Guandu watershed (mean sequence length 4776 bp), and 5185 contigs from drinking water (mean sequence length 3255 bp) were analyzed. Eighteen distinct subsystems, containing genes attributed to resistance and adaptation to antimicrobials, metals, and other environmental pollutants, were distributed among all samples, according to analyzes at the MG-RAST database. While the Guandu watershed showed the greatest diversity of subsystems (*n* = 16), São João exhibited the least diversity (*n* = 4) while samples of drinking water revealed 12 subsystems.

**Figure 4:**
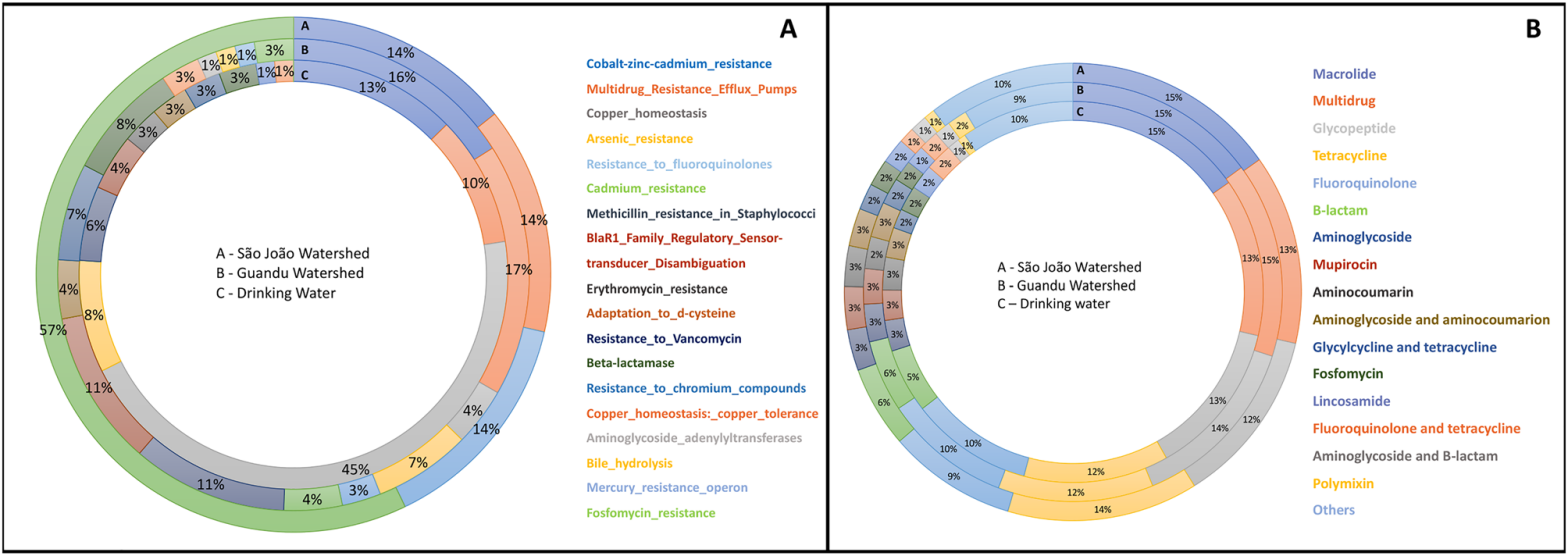
(**A**) Relative abundance of sequences related to antimicrobial resistance subsystems and toxic compounds in grouped samples of São João watershed, Guandu watershed, and Drinking water; (**B**) Relative abundance of ARGs concerning antimicrobial classes, in the three groups of samples.

Fifty-seven percent of São João watershed sequences and 4% of Guandu sequences were attributed to *Cadmium_resistance*; this system was not found in drinking water samples. The *Cobalt-zinc-cadmium_resistance* subsystem, including zinc, cobalt, and cadmium efflux systems encoded mainly by the *cusA* gene and the *czc* operon, was found in 14% of the São João watershed, 16% of Guandu watershed, and 13% of drinking water sequences. The presence of genetic determinants of the *Multidrug_Resistance_Efflux_Pumps* subsystem was also revealed in the three sample groups (14% São João watershed, 17% Guandu watershed, 10% drinking water) mainly composed of members of the Multidrug And Toxic compound Extrusion (MATE) family in the São João and Guandu watersheds samples (> 90%). In the drinking water samples, the MATE family (37.5%), the superfamily pumps of efflux resistance-nodulation-cell division (RND) encoded by the *cmeA* gene (12.5%), and the *macA*/*macB* macrolide efflux system (25%) were found (Fig. 4a).

Through the CARD database searches, 4881 antimicrobial resistance genes were annotated from the Guandu watershed, 3705 from the São João watershed, and 3,385 from the drinking water samples. This analysis revealed the prevalence of three types of resistance mechanisms: antibiotic efflux, antibiotic target alteration/protection, and antibiotic inactivation, evenly distributed among the samples (Fig. 4b).

In general, ARGs found in the samples encode many types of proteins and enzymes capable of conferring resistance to antimicrobials. The genes capable of conferring resistance to 4 or more classes of antimicrobials were grouped as Multidrug, while the less abundant antimicrobial classes (< 1%) were grouped as Others. Resistance genes to macrolides were prevalent in all three sampling sites (15.2% São João, 14.9% Guandu, and 15.3% drinking water), followed by ARGs against glycopeptides, tetracyclines, fluoroquinolone, β-lactams, and others. The most abundant genes in both watersheds and drinking water samples were *macB* and *tetA*(58), both associated with efflux systems. It is worth noting the presence of the genes bla_AIM_ (Guandu and São João watersheds), bla_GIM_ (drinking water, Guandu and São João watersheds), bla_IMP_ (Guandu watershed), and bla_VIM_ (drinking water, Guandu, and São João watersheds) encoding metallo-β-lactamase enzymes. Even more surprising, the presence of the *mcr-3* (Drinking water, Guandu and São João watersheds), *mcr-4* (Guandu watershed), and *mcr-9* (Drinking water and Guandu watershed) genes that can provide resistance to colistin and are currently considered a serious public health issue, was also observed.

## Discussion

The consumption of contaminated drinking water is a major pathway for environmental ARB to enter into the human gut^22^. The São João and Guandu watersheds are frequently impacted by elevated levels of chemical and fecal pollution, due to a lack of sewage treatment in the surrounding cities. Therefore, this study aimed to determine the presence of antimicrobial residues in water resources and to evaluate their possible impacts on the microbial resistome. Antimicrobial residues in the aquatic environment may originate from hospital, domestic, rural effluents (aquaculture, livestock)^23,24^, and the pharmaceutical industry^25^.

In this study, substances from the β-lactam, macrolide, and sulfonamide antimicrobial classes were detected in samples of untreated waters, being the Guandu watershed the one with the highest rates of antimicrobials. Clarithromycin, azithromycin, and sulfamethoxazole have also been found in drinking water samples. It is worthy to noting that clarithromycin residues were observed in all three environments, as well as a high proportion of resistance genes to this antibiotic, suggesting an impact of the drug on the spread of resistance genes^26^. In addition, troleandomycin and roxithromycin were detected only in drinking water samples. Macrolides, such as clarithromycin and azithromycin, are widely administered in human and animal medicine and can be carried to water resources through agricultural soil and the application of sewage sludges or fertilizers^27^.

Many antimicrobials have already been detected in drinking water in several developed countries at levels generally < 100 ng L^−1^^28^. High levels of carbamazepine, clofibric acid, and sulfamethoxazole have also been observed in drinking water in countries in Europe and North America^29^. However, our data showed that although cephalexin was found in 60% (6/10) of the samples from Guandu and 20% (2/10) from São João in levels ranging between < 10 ng L^−1^ and > 500 ng L^−1^ it was not detected in drinking waters. Some antimicrobials can be eliminated via abiotic or biotic degradation, but their continued introduction can make them pseudo persistent in aquatic environments^29^. The presence of antimicrobials in drinking water is due to their incomplete removal during conventional treatment steps in WWTPs. In addition, antimicrobial residues can accelerate the emergence and evolution of ARB and ARGs in the environment^30^.

Water also represents an important way of spreading bacteria between different aquatic environments, including freshwater habitats that harbor the richest bacterial diversity^31^. It is well known that heterotrophic prokaryotes play important roles in the structure and dynamics of trophic networks and the remineralization of organic matter^32^. We find that most of the environments analyzed (93.5%; 29/31) are dominated by the phyla *Proteobacteria*, *Actinobacteria*, and *Bacteroidetes*. *Proteobacteria* have great metabolic diversity, a fact that allows their dissemination in the most varied environments^33^.

A relevant finding of our study was the high abundance of *Actinobacteria* in the Juturnaíba Dam, which is one of the groups best known for containing organisms that produce antimicrobials and carriers of MDR profiles, and one of the most prevalent sources of ARGs^34^. *Actinobacteria* have many acetyltransferases and phosphotransferases, which represent the greatest resistance mechanism to aminoglycosides^34^.

Physical, chemical, and biological pollution can influence the microbial composition, with potential effects on water quality and safety. Although maintaining a safe and reliable supply of drinking water is of critical importance, few potentially pathogenic microorganisms are recognized and even less are regulated^35^. The homogeneity between the bacterial communities showed in our study can be explained by the urbanization process which leads to the discharge of high loads of untreated waste and sewage containing feces and xenobiotic compounds into bodies of water^36,37^.Environments that are not impacted have a higher prevalence of phyla Acidobacteria and Verrucomicrobia^36^. Also, the treatment of drinking water aims to reduce the microbial load, which explains why alpha and beta diversities of the microbial communities of untreated watershed waters were higher compared to the drinking water communities^38,39^.

Our plasmidome analyses revealed a high abundance of the czc (cobalt-zinc-cadmium) efflux system in all environments analyzed. This system is strongly associated with locations impacted by oil, sludge, metals, and other urban waste^40^. There is great concern about the relationship between this system and the increase in antimicrobial resistance. *P. aeruginosa* strains isolated from urinary catheters, susceptible to carbapenems and carrying the czc operon, have demonstrated resistance to imipenem, when exposed to zinc. Also, analysis of cross-resistance mechanisms revealed co-regulation of *czcR* overexpression and a decrease in *oprD* expression, that encodes a channel associated with resistance to carbapenems, especially imipenem^41,42^.

It is already known that ARBs can survive the selective pressures that occur during the water treatment process^43^. Meanwhile, the removal of ARGs varies depending on the water treatment scheme. Chlorine disinfection can eliminate many ARBs but does not destroy ARGs resulting in their discharge into aquatic environments^44,45^.

Several ARGs detected in our study have been previously reported in environmental waters, sediments and soils, drinking water, and WWTP^46,47^. The *bla*_*NDM*_ and *bla*_*CTX-M-type*_ genes often found on mobile genetic elements such as plasmids, have already been reported in drinking water globally^22,48^. We also show the presence of several genes encoding MBLs (metallo-β-lactamases), carbapenemases, and the *mcr* (mobile colistin resistance) genes, which confers resistance to colistin, associated with plasmids.

It is noteworthy that, as far as we know, this is the first study reporting the presence of the *mcr-3* and *mcr-9* genes in drinking water samples. Some *mcr-like* genes have already been described in water systems, such as *mcr*-1 which, although it was first described in *Enterobacteriaceae* isolated from animals, food, and humans in China^49^, has already been revealed in China's water systems^46^. So far, the presence of the *mcr-9* gene has not been described in Brazil, either in bacterial isolates or through metagenomic studies. Although colistin residues were not revealed in our study, the occurrence of *mcr*-like genes in the Guandu and São João watersheds and drinking water samples could be related to the low levels of colistin and/or other drugs important for spreading of *mcr* resistance genes in those environments. Stanton et al.^50^, demonstrated evidence that antibiotics in low concentrations (below minimum inhibitory concentrations) promote the emergence and persistence of antibiotic resistance in natural environments. In fact, colistin has been heavily added to animal feed, as a growth promoter in cattle, pigs, and poultry, in Brazil^51^. The same was observed in some Asian countries, including China, India, Japan, and Vietnam where colistin is widely used to improve weight gain in animals^52^. In Europe, it is mainly used to treat infections caused by *Enterobacteriaceae* in pigs, chickens, cows, sheep, and goats^53^.

The β-lactamase enzyme production is the most common mechanism of bacterial resistance to β-lactam antimicrobials^53^. In this study, the genes encoding carbapenemases were found only in one of the evaluated watersheds (Guandu). However, Gram-negative bacteria carrying genes for resistance to carbapenems have already been isolated from samples of rivers, wastewater, and drinking water, emphasizing their high potential for dissemination in the environment^54,55^.

In addition to other carbapenem resistance genes, the *bla*_GIM_ and *bla*_VIM_ genes were found in the drinking water samples in the present study. In general, strains carrying MBL genes are MDR which are a serious therapeutic problem in clinical isolates. The description of genes encoding MBLs associated with MGEs has considerably increased attention to these enzymes, including them among the main threats to human health for the twenty-first century^56,57^.

Widespread antimicrobial resistance represents a serious threat to human health since it is associated with the loss of the therapeutic potential of antibiotics and the consequent morbidity and mortality^58^. Currently, studies suggest that chemical compounds that are not antimicrobial can also select and stimulate antimicrobial resistance, such as heavy metals^59^, disinfectants^60^, disinfection by-products^61^, and nanomaterials^62^.

Treatment of drinking water in treatment plants is not aimed at removing disinfectants, and quite often, a residual amount is maintained in the supply system to prolong water quality during distribution^63^. However, the consequences and selective pressures of such residuals are not usually considered regarding the presence of ARGs, ARBs, and MGEs. Besides that, these plants do not efficiently remove antimicrobials and metals^64,65^. Hence, this selection pressure caused by disinfection, antimicrobials and metallic agents could continue throughout distribution, and bacteria carrying resistance determinants, or capable of acquiring them, may persist in the drinking water^11,66^.

Current regulations do not establish the monitoring and control of ARBs, ARGs, and MGEs in drinking water and wastewater. Our data emphasize the importance of introducing measures to reduce the disposal of antibiotics and other pollutants capable of promoting the enrichment and maintenance of the microbial resistome. Besides that, our data indicate that mitigation strategies should be put in place to reduce the risk of AMR and to prolong the efficacy of the currently available antimicrobial agents for use in animals and humans.

## Materials and methods

### Samples collection

Five collection sites were selected in Guandu Watershed (22°50′22.11″ S and 43°36′36.70″ O) (Queimados, Guandu, Piraí and Macacos rivers and Guandu Dam), and five sites in São João Watershed (22°37′36.60″ S and 42°17′54.36″ O) (Capivari, Bacaxá and São João rivers, São João river mouth and Juturnaíba Dam). Samples (5 L from each site) were collected in sterile bottles, six months apart (January and June/2015). In addition, in January/2015, 11 household tap drinking water samples (5 L from each point) were collected in different neighborhoods (one sample per neighborhood) from Rio de Janeiro city (Centro, Copacabana, Ilha do Governador, Jacarepaguá, Jardim Botânico, Leblon, Realengo, Santa Teresa, Vista Alegre) and from the small towns of Unamar and Itaguaí (one sample per city) in Rio de Janeiro State. All samples were collected in three replicates and refrigerated until processed in the laboratory within 24 h. All experiments were performed using kits and internal controls. Metadata for all samples is included in supplementary Table 2.

All drinking water is provided by Guandu Water Treatment Plant (GWTP). The GWTP is in the Guinness Book as the world's largest drinking water treatment plant in continuous production with a flow rate of about 45,000 L per second^67^. Upon reaching the GWTP, a chemical coagulant is added to the water, followed by a polyelectrolyte. With the coagulant adequately dispersed, the water passes through hydraulic flocculators, whose controlled agitation promotes the collision of the particles and consequently the agglutination, forming the flocs. The water then enters the sedimentation tanks (decanters), where the speed is reduced, and the flakes already formed and with greater weight sink to the bottom. The clarified water is collected through channels on the blade’s surface and distributed to the filtration system. The filters are composed of layers of sand with a granulometry capable of retaining the finest particles that are still present in the clarified water. After being filtered, the water flows to the contact reservoirs, where disinfection occurs with the addition of chlorine. After being disinfected, the water is fed through underground channels to the high-pressure lifts. In these channels, the pH correction occurs with the addition of quicklime. Fluoride is also applied to treated water as an auxiliary agent in the fight against dental caries^68^.

### Antimicrobial residues detection

#### Chemicals and materials

Amoxicillin tryhidrate (AMOX), ampicillin (AMPI), cefaclor (CFCL), cefadroxil (CFDX), cefalexin hydrate (CFLX), cefazolin (CFZL), clarithromycin (CLA), ciprofloxacin hydrochloride (CPF), norfloxacin (NOR), tetracycline hydrochloride (TC) and sulfamethoxazole (SMZ) were chemical reference substances from the Brazilian Pharmacopeial Convention (Santa Maria, RS, Brazil). Azithromycin dehydrate (AZI), roxithromycin (ROX), spiramycin (SPI), oleandomycin (OLE), tilmicosin (TILM), and cefquinome sulphate salt (CFQN) were obtained from Dr. Ehrenstorfer (Augsburg, Germany). Oxytetracycline (OTC), doxycycline hyclate (DC), hydrochloride salts of chlortetracycline (CTC) and demeclocycline (DMC), dapsone (DAP), sulfacetamide (SCT), sulfadimethoxine (SDM), sulfamerazine (SFM), sulfamethazine (SMT), sulphaquinoxaline (SQN), sulfathiazole (STZ), tylosin tartarate (TYL), troleandomycin (TRO), erythromycin (ERY), cephapirin sodium salt (CPPN), ceftiofur (CFTF), cefoperazone (CFPZ), benzylpenicillin sodium salt (PENG), oxacillin sodium salt hydrate (OXA), moxifloxacin (MXF) and ofloxacin (OFX) were supplied from US Pharmacopeial Convention (Rockville, MD, USA). Phenoxymethylpenicillin potassium salt (PENV), cloxacillin sodium salt hydrate (CLOX), dicloxacillin sodium salt hydrate (DCLOX) and nafcillin sodium salt (NAFC) were supplied from WHO Collaborating Centre for Chemical Reference Substances (Stockholm, Sweden). Methacycline (MTC), 4-epioxytetracycline (4-EOTC), 4-epitetracycline (4-ETC) and 4-epichlortetracycline hydrochloride (4-ECTC) were acquired from Acros (Pittsburgh, PA, USA). Ampicillin-d5 (AMPID5) was purchased from Purity Grade Standards (San Francisco, CA, USA). Desacetylcephapirin (DESAC) was supplied from Bristol-Myers Squibb (New York, USA).

Methanol (MeOH) and acetonitrile (ACN) HPLC grade, hydrochloric acid (HCl) and formic acid (FOA) analytical grade were purchased from Merck (Darmstadt, Germany). Sodium hydroxide (NaOH), acetone (ACE) and ascorbic acid (ASA) were purchased from Merck (Darmstadt, Germany). Ethylenediaminetetracetic acid disodium dihydrate (EDTA) was acquired from Calbiochem (Gibbstown, NJ, USA). Ultrapure water was obtained from a Milli-Q purification system (Millipore, Bedford, MA, USA).

Solid-phase extraction (SPE) was performed with 60 mg Oasis® HLB cartridges from Waters Corp. (Milford, MA, USA). Membrane filters of polyvinylidene fluoride (PVDF) with pore size 0.22 µm were purchased from Millipore (Billerica, MA, USA).

#### Preparation of standard solutions

The stock standard solutions were prepared to obtain a concentration of approximately 1000 μg mL^−1^. Stock solutions of β-lactams (BL) were prepared in water while those of fluoroquinolones (FQ) in a 0.03 mol L^−1^ NaOH. Finally, macrolides (MC), sulfonamides (SF) and tetracyclines (TC) solutions were prepared in MeOH. The amount weighed for each standard was calculated considering purity, water content and free acid/basic corrections. The solutions were transferred to microtubes and stored in a freezer at − 70 °C or below. DMC and AMPID5 were used as internal standards.

Intermediate and working standard solutions were freshly prepared at several concentrations by appropriate dilution of stock standard solutions.

#### Analytical method

The extraction methodology for antimicrobial residues was based on the standard method from the United States Environmental Protection Agency (US EPA)—Method 1694^69^, with modifications described by Monteiro et al.^70^.

Samples were previously filtered through filter paper and 0.22 µm PVDF membrane filter. A 50 mL aliquot of each sample was spiked with 100 ng L^−1^ of the internal standards, acidified to pH 2.5 with HCl, and 2 mL of 25 mg L^−1^ EDTA stock solution was added. For drinking water samples, 2 mL of 625 mg L^−1^ ASA was added to reduce any residual chlorine. This solution was applied to an Oasis® HLB cartridge that had been previously conditioned in sequence with 3 mL of MeOH, 3 mL of ultrapure water and 3 mL of ultrapure water acidified to pH 2.5 with HCl. After being washed twice with 2 mL of water, SPE cartridges were vacuum-dried (− 35 kPa) for 2 min. Antimicrobials were eluted with three portions of 2 mL MeOH and one portion of 2 mL ACE, using gravity flow only. 4 mL aliquots of the eluate were transferred to two centrifuge tubes and evaporated to dryness with N_2_ in a temperature up to 47 °C. The residues were reconstituted with 1 mL of 0.1% FOA:MeOH (80:20, v/v) for TC and SF analysis and 1 mL of MeOH:H_2_O (65:35, v/v) for BL, MC and FQ analysis, vortexed for 30 s and filtered through a 0.22 µm polyvinylidene fluoride (PVDF) syringe filter into amber auto-sampler vials.

The chromatographic analysis was performed on a Shimadzu Prominence HPLC (Kyoto, Japan) equipped with a quaternary pump (LC-20AD), a membrane degasser (DGU-20A5), an auto-sampler (SIL-20AC), a column oven (CTO-20AC) and a system controller (CBM-20A) interfaced to a triple quadrupole mass spectrometer (API5000, Applied Biosystems/MDS Sciex, Foster City, CA, USA) with the TurboIonSpray® source. Analyst® V1.4.2 LC/MS control software was used. The analytical column was a Pursuit™ C18 RS (100 mm × 2 mm i.d., 3 µm particle size, 200 Å), with a respective guard column (Varian, Lake Forest, CA, USA). Mobile phases A, B and C were prepared using water, ACN and MeOH, respectively, all with 0.1% FOA. A gradient elution program for TC and SF method was used with a flow rate of 0.15 mL min^−1^ at 25 °C and for BL, MC and FQ another gradient elution was used with a flow rate of 0.30 mL min^−1^ at 35 °C. The injection volume was 25 µL for both methods. The auto-sampler was set at 4 °C. Positive electrospray ionization technique (ESI +) in Multiple Reaction Monitoring (MRM) acquisition mode was used to monitor two ions for each substance.

A six-point calibration set was freshly prepared by spiking varying levels of working standard solutions in ultrapure water. The analytical curves for all analytes in the concentration range from 25 to 1000 ng L^−1^ were constructed in order to quantify the analytes in samples.

The chromatographic peaks were integrated with the IntelliQuan algorithm of the Analyst® software. A signal-to-noise ratio of the peaks equal or greater than 3:1 for at least 2 transitions was required for detection. Relative retention times and relative abundances between quantification and confirmation MRM transitions in both matrix-fortified standards and samples were used as confirmation criteria according to recommended tolerances in 2002/657/EC Commission Decision, that was in place when this work was carried out^71^. Samples were considered contaminated when analytes were detected according to identification criteria by liquid chromatography tandem mass spectrometry and the concentration values exceeded the limits of detection (LOD).

### Bacterial community composition

Water samples were filtered through 0.22 µm cellulose acetate membranes (Millipore, USA) under aseptic conditions. All experiments were performed using kits and internal controls. DNA was extracted from the filters using the PowerWater DNA Isolation Kit (Qiagen Science, USA). For the preparation of the amplicon library, the DNA was quantified using a Qubit 2.0 Fluorometer (ThermoFisher Scientific, USA), and samples were diluted to achieve the concentration of 5 ng μL^−1^ per sample. The V4 hypervariable region of the 16S rRNA gene was amplified by PCR using the primers 16Sf (5′- GTGCCAGCMGCCGCGGTAA-3′) and 16Sr (5′- GGACTACHVGGGTWTCTAAT-3′) with the appropriate barcodes and adapters^72^. PCR products were purified using the ChargeSwitch™ PCR Clean-Up Kit (ThermoFisher Scientific, USA). Each individual sample library was diluted to 4 nM and then pooled and paired-end sequenced on a MiSeq system (Illumina Inc. USA), using the 500 cycles MiSeq Reagent v2 Kit.

Quality analysis of raw reads was carried out with FastQC software^73^ and the filtering of sequences with an average quality equal to or greater than 20 was performed by the PRINSEQ program^74^. Data analysis was performed using QIIME (Quantitative Insights Into Microbial Ecology) 1.9.1^75^. The data were compared with the SILVA Ribosomal RNA database (non-redundant) 132 release^72^ with a maximum e-value of 1e-5, and a minimum identity of 99%, which generated a table with taxonomic groups. Statistical analyzes such as Alpha and Beta diversity were calculated using the MicrobiomeAnalyst web platform (https://www.microbiomeanalyst.ca/)^76,77^. The diversity of the bacterial communities was assessed using the Chao1 index and Simpson's index calculated for OTUs with the evolutionary distance of 0.01 (or 99% 16S rRNA gene sequence similarity). Principal coordinate analysis (PCoA) between bacterial communities present in the Guandu, São João watersheds and in drinking water was built using the Jaccard method with PERMANOVA and using the bacterial OTUs.

### Plasmidome

Plasmid DNA (pDNA) was extracted from the filters by alkaline lysis using the *Plasmid Mini Kit* (Qiagen Science, USA) according to the manufacturer's protocol. pDNA was precipitated with isopropanol and washed with 70% ethanol. To remove possible traces of genomic DNA the precipitate was treated with ATP-dependent Plasmid Safe DNase (Epicentre, USA) according to manufacturer's instructions^46^. The pDNA was quantified using a Qubit 2.0 Fluorometer (ThermoFisher ScientificTM, USA) according to the manufacturer's manual.

A pDNA sequencing library was prepared using the Nextera XT DNA Library Prep Kit (Illumina Inc. USA) following the manufacturer's recommendations. Paired-end sequencing was performed with the 600 cycles Miseq Reagent Kit v.3 on the MiSeq platform (Illumina Inc. USA). Sequence quality checks were performed with the FastQC software^73^ and sequence filtering with an average quality of 20 or higher was performed by PRINSEQ^78^. Contigs were assembled with SPAdes version 3.13^79,80^ using the metaSPAdes pipeline.

The sequences were analyzed by the MG-RAST (Meta Genome—Rapid Annotation using Subsystem Technology) platform^81^, where the annotation can be viewed in several different categories, including subsystems. A subsystem can be understood as a set of functional roles that implement a certain biological or structural process^82^. The subsystems are classified into hierarchical levels, so that level 1 includes general catabolic and anabolic functions (for example, DNA metabolism), and levels 2 and 3 contain more specific pathways, such as antimicrobial resistance and other compounds^83^.

In addition, the sequences were compared against the Comprehensive Antibiotic Resistance Database (CARD) database^84,85^ with DIAMOND^86^. Only alignments with an e-value < 1e5, coverage > 60% and amino acid identity > 30% were considered^87^.

### Ethical approval

This article does not contain any studies with human or animals performed by any of the authors.

## Supplementary Information

Supplementary Information 1.

Supplementary Information 2.

## Data Availability

All data employed in this report are available in GenBank under BioProject PRJNA812588 (https://www.ncbi.nlm.nih.gov/bioproject/PRJNA812588). In addition, metagenome sequence data are available on MG-RAST under accession numbers mgm4919709.3, mgm4919786.3, mgm4919818.3.
